# High Resolution Melting analysis as a rapid and efficient method of screening for small mutations in the *STK11* gene in patients with Peutz-Jeghers syndrome

**DOI:** 10.1186/1471-2350-14-58

**Published:** 2013-05-30

**Authors:** Pawel Borun, Anna Bartkowiak, Tomasz Banasiewicz, Boguslaw Nedoszytko, Dorota Nowakowska, Mikolaj Teisseyre, Janusz Limon, Jan Lubinski, Lukasz Kubaszewski, Jaroslaw Walkowiak, Elzbieta Czkwianianc, Monika Siolek, Agnieszka Kedzia, Piotr Krokowicz, Wojciech Cichy, Andrzej Plawski

**Affiliations:** 1 Institute of Human Genetics, Polish Academy of Sciences, Strzeszyńska 32, Poznan, 60-479, Poland; 2 Department of General, Gastroenterological and Endocrinological Surgery, Poznan University of Medical Sciences, Poznan, Poland; 3 Department of Dermatology, Venereology and Allergology, Medical University of Gdansk, Gdansk, Poland; 4 Maria Sklodowska-Curie Memorial Cancer Centre and Institute of Oncology, Warsaw, Poland; 5 Department of Gastroenterology, Hepatology and Immunology, Children's Memorial Health Institute, Warsaw, Poland; 6 Department of Biology and Genetics, Medical University of Gdansk, Gdansk, Poland; 7 International Hereditary Cancer Center, Department of Genetics and Pathology, Pomeranian Medical University, Szczecin, Poland; 8 Fourth Clinical Hospital, University of Medical Sciences, Poznan, Poland; 9 First Chair of Pediatrics, Department of Pediatric Gastroenterology and Metabolic Diseases, University of Medical Sciences, Poznan, Poland; 10 Department of General and Colorectal Surgery, Poznan University of Medical Sciences, Poznan, Poland; 11 Department of Gastroenterology, the Polish Mother's Memorial Hospital, Research Institute in Lodz, Lodz, Poland; 12 Holy Cross Oncology Center, Kielce, Poland

## Abstract

**Background:**

Peutz-Jeghers syndrome (PJS) is a rare hereditary syndrome characterized by the occurrence of hamartomatous polyps in the gastrointestinal tract, mucocutaneous pigmentation and increased risk of cancer in multiple internal organs. Depending on the studied population, its incidence has been estimated to range from 1:200 000 even up to 1:50 000 births. Being an autosomal disease, PJS is caused in most cases by mutations in the *STK11* gene.

**Methods:**

The majority of causative DNA changes identified in patients with PJS are small mutations and, therefore, developing a method of their detection is a key aspect in the advancement of genetic diagnostics of PJS patients. We designed 13 pairs of primers, which amplify at the same temperature and enable examination of all coding exons of the STK11 gene by the HRM analysis.

**Results:**

In our group of 41 families with PJS small mutations of the STK11 gene were detected in 22 families (54%). In the remaining cases all of the coding exons were sequenced. However, this has not allowed to detect any additional mutations.

**Conclusions:**

The developed methodology is a rapid and cost-effective screening tool for small mutations in PJS patients and makes it possible to detect all the STK11 gene sequence changes occurring in this group.

## Background

Peutz-Jeghers syndrome (PJS, MIM # 175200) is a rare, hereditary predisposition characterized by the presence of hamartomatous polyps in the gastrointestinal tract, occurrence of lentigines on mucous membranes (mainly on lips, oral cavity and nostrils) as well as by an increased risk of malignant tumours of various internal organs [1-5]. Depending on the population examined, the incidence of the syndrome is estimated to fluctuate from 1:200 000 even up to 1:50 000 of births [2].

PJS is an autosomal dominant disease caused by mutations in the *STK11* gene (MIM # 602216) located on the small arm of chromosome 19 at position 19p13.3. This gene comprising 22 637 base pairs consists of 10 exons, nine of which contain a coding sequence of 48.6 kDa protein made up of 433 amino acids, which fulfils the function of serine-threonine kinase. The *STK11* is a suppressor gene, and the kinase encoded by it, plays an important role in cell metabolism regulation, proliferation and apoptosis [6,7].

Until recently, over 230 different *STK11* gene mutations causing the Peutz-Jeghers Syndrome have been described in the Human Gene Mutation Database (HGMD) (http://www.hgmd.cf.ac.uk; the state on 08. 2012). Most of them are small mutations including: 72 point missence/nonsense mutations, 26 mutations in splicing sites as well as 102 small insertions, deletions and indel type mutations. The remaining 31 mutations described concern rearrangements of larger fragments of the *STK11* sequence including 25 large deletions, 3 large insertions and 3 combined mutations.

In molecular studies of the *STK11* gene in patients with the PJS, mutations are identified in almost all cases of persons with a positive family history and in approximately 50-90% when only patients who met the clinical criteria for PJS were considered [8,9]. Differences in percentage proportions of the identified mutations for individual patient groups in investigations carried out in different parts of the world can be attributed both to the specificity of the population and the way of patient selection for examinations as well as the applied method of research. Recent studies revealed that copy number variations (CNV) can be the cause of a considerable proportion, even up to 30%, of PJS cases; this can explain the lower percentage of mutations detected in patient groups not tested with methods able to identify large rearrangements (e.g. MLPA or qPCR) [8,10-12]. However, small mutations still constitute the highest percentage of changes causing the disease and, therefore, the designing of a rapid and cost-effective method of their detection is a key aspect in the advancement of genetic diagnostics of PJS patients.

One of the more novel techniques employed in screening investigations, which allows identification of small sequence changes within the examined fragments is the high resolution melting (HRM) analysis. The mode of action of the method is based on monitoring the behaviour of DNA fragments, amplified earlier during the denaturation process. The analysis is possible due to the presence in the reaction mixture of a fluorescent dye which intercalates only the double-stranded DNA sending a strong fluorescent signal allowing to observe the melting process, i.e. conversion of dsDNA into ssDNA in the course of denaturation. Comparison of melting profiles of individual fragments with one another makes it possible to select those that indicate differences in the course of denaturation, which at the same time reflect changes in the sequence. HRM is a screening technique and fails to provide accurate information about changes in the sequence and, therefore, all atypical melting profiles require confirmation by sequencing. Nevertheless, high sensitivity of the method, which according to some researchers reaches even 100%, allows considerable reduction of sequencing numbers and, consequently, of the cost of analyses, at the same time maintaining and sometimes even increasing the identification effectiveness of mutations in comparison with earlier screening methods [13,14].

The objective of our investigations was to develop a rapid and effective detection methodology of small mutations in patients suffering from PJS. Bearing in mind the heterogenous nature of mutations in the *STK11* gene, the size of gene exons and effectiveness of individual screening techniques, we decided to develop a methodology based on the HRM technique.

## Methods

The material for investigations was the DNA obtained from 41 probands with diagnosed Peutz-Jeghers syndrome. All patients included in this study fulfilled the diagnostic criteria of PJS (histopathologically confirmed hamartomatous polyps and mucocutaneous pigmentation). The DNA was isolated from peripheral blood and for this purpose the method of extraction with phenol and chloroform was used, which was followed by purification by means of the method of precipitation with ethanol.

In accordance with the standards adopted for HRM, primers were designed using the Primer3plus (http://www.bioinformatics.nl/primer3plus/) software [15]. Each of the designed primer pairs was checked with respect to the melting profile. In addition, PCR product heterogeneity on agarose gel (1.5% agarose gel, electrophoresis – 30 min., voltage – 100 V) was also checked. On the basis of these analyses, 13 primer pairs were selected which allowed examination of all *STK11* gene exons together with sequences of the exon-intron junctions. The obtained PCR amplicons were contained within the length range of 150–250 bp, while primers annealing temperature was 60°C for all pairs. Primers sequences are collated in Table 1. Fragments were amplified using Type-it HRM kit [Qiagen] and the analysis was performed on a Rotor-Gene Q equipment [Qiagen]. The PCR was performed according to the manufacturer’s instructions. PCR conditions were: 95°C for 5 minutes, followed by 40 cycles of pre-incubation at 95°C for 10 seconds, annealing for 30 seconds at 60°C and extension at 72°C for 10 seconds. The HRM step was performed from 60°C to 95°C raising the temperature by 0.1°C degree at each step. The obtained plots were analyzed using Rotor-Gene Software: 2.0.2.

**Table 1:** List of primers utilised in the HRM analysis

**Amplicon**	**Forward primer**	**Reverse primer**	**Product length [bp]**	**Annealing temperature [°C]**
1a	GGAAGTCGGAACACAAGGAA	CATCAGGTACTTGCCGATGA	231	60
1b	CTCCACCGAGGTCATCTACC	CCCCAGCAAGCCATACTTAC	221	60
2	AGGCCATCATCCTGACGTT	CCCAACACCGGAAAGGATA	159	60
3	CTCCCTGGGCCTGTGAGT	CCCTGCCCCGCGCACGCA	165	60
4a	CAGGACGGGTGTGTGCTG	CCCTAGCACGTGCCTACCT	175	60
4b	GCCTGGAGTACCTGCATAGC	GTGCAGCCCTCAGGGAGT	172	60
5a	CACTCCCTGAGGGCTGCAC	GGGGCACTTACAGGGTGAC	180	60
5b	GGCTTCAAGGTGGACATCTG	CTGTGGCCAGAGAGGGTCT	161	60
6	TCAACCACCTTGACTGACCA	ACCCCCAACCCTACATTTCT	249	60
7	CCTTAGGAGCGTCCAGGTATC	CTCAACCAGCTGCCCACAT	244	60
8a	CTGGGTCGGAAAACTGGAC	GTGCAGGTCCTCCAAGTACG	182	60
8b	GAGCCCAGACACCAAGGAC	GACATCCTGGCCGAGTCA	188	60
9	TCAGCTCAGGCCACACTTGC	AGCCTCACTGCTGCTTGC	232	60

All samples were analyzed in two separate runs in the presence of at least three wild type controls sequenced earlier. Amplicons exhibiting a melting profile different from wild type controls in the course of HRM were selected and subjected to sequencing. In cases in which no evident pathogenic mutations were detected during HRM analyses, sequencing of the entire *STK11* gene coding sequence was performed. The PCR products were purified by precipitations and sequenced on both strands using the Big Dye Terminator Cycle Sequencing Kit v3.1 Kit (Applied Biosystems). The sequence products were purified and separated on an automated sequencer (ABI 3130xl Genetic Analyzer, Applied Biosystems).

Standard nomenclature (http://www.hgvs.org/mutnomen/webcite) was used to describe sequence variations with +1 corresponding to the A of the ATG translation initiation codon of GeneBank NM_000455 for *STK11*.

## Results

**Figure 1:**
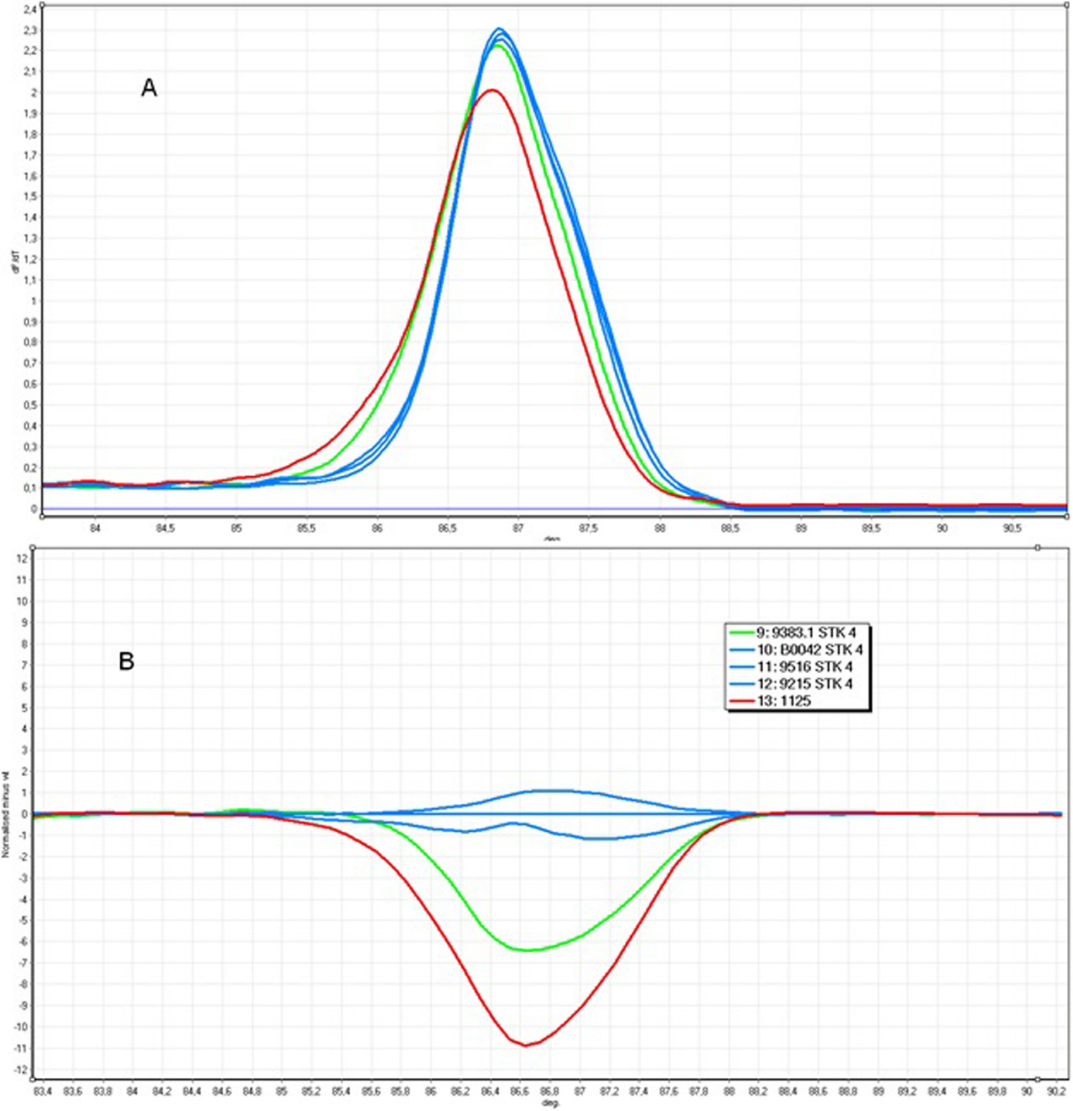
**The example HRM analysis of exon 4 of the *STK11 *gene; green curve - c.493G > T, red curve - c.488G > A, blue curve - wild type; A. Melt profile B. HRM analysis.**

**Figure 2:**
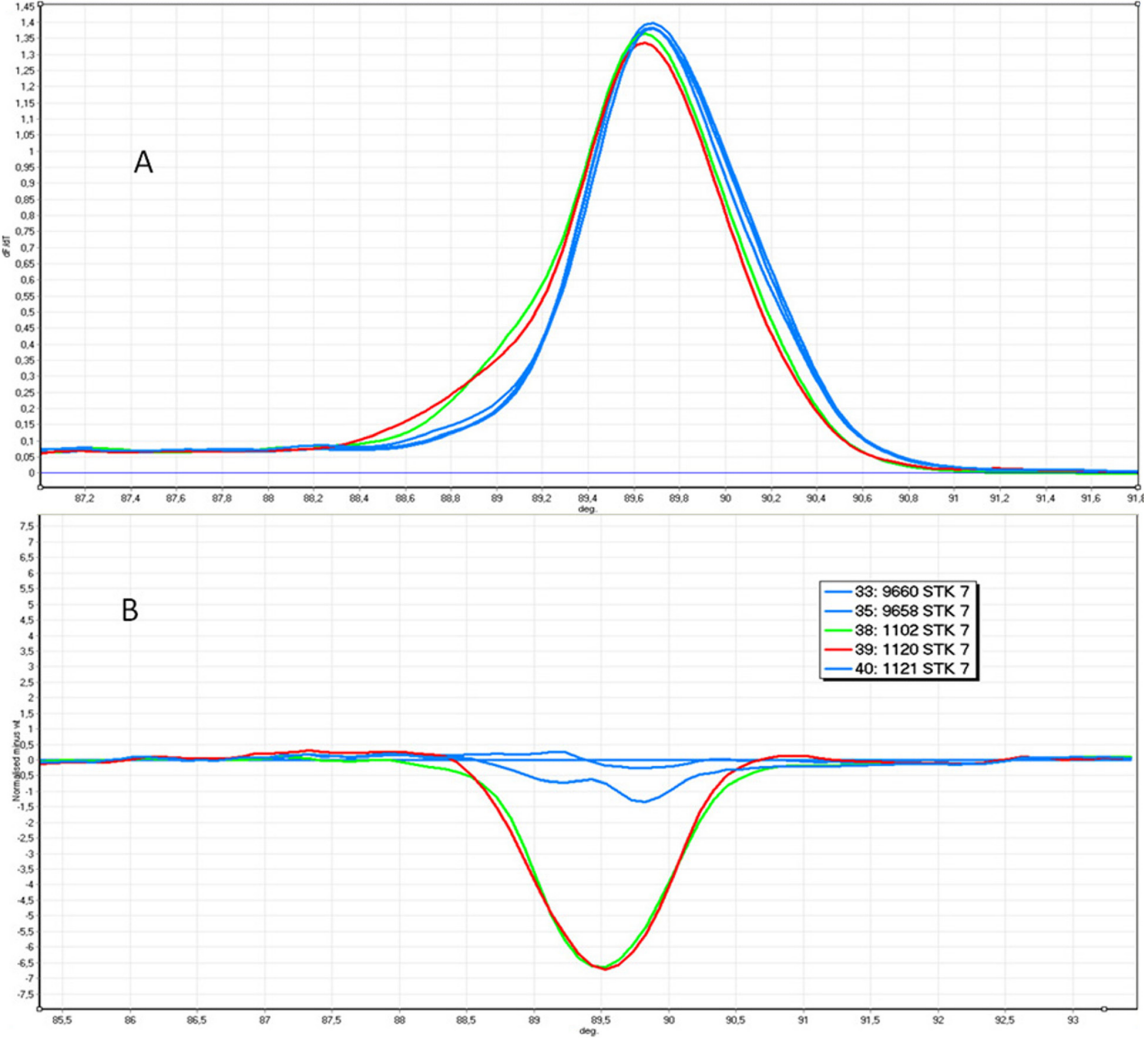
**The example HRM analysis of exon 7 of the *STK11 *gene (1nt deletion and SNP class III); green curve - c.910delC, red curve - c.876C > G, blue curve - wild type; A. Melt profile B. HRM analysis.**

Small *STK11* gene mutations were detected in 22 families (54%) seven of which were novel, so far undescribed changes (Table 2). Mutations were detected in all gene coding exons with the exception of exons 3 and 9. Distinct disproportions in the distribution of mutations were apparent. A majority of the detected changes occurred in exons 1, 4 and 7. The examination of exon 1 revealed the presence of 4 kinds of mutations of which two were novel ones, so far undescribed in literature (c.160_161insC, c.178delT). The third change was the earlier observed nonsense mutation c.180C > G the consequence of which was the appearance of the premature stop codon in position 60 (p.Tyr60X). The fourth mutation in this exon was c.157_158dupG which creates a termination site after the next 110 codons (p.Asp53Glyfsx110). Five kinds of mutations (c.474delT, c.481A > T, c.488G > A, c.493G > T, c.580G > A) were detected in exon 4 in seven of the examined families (Figure 1). The most frequent mutation (detected in 3 families) was the G > A substitution in position 580 resulting in a p.Asp194Asn amino acid substitution. The performed analysis of exon 7 revealed the presence of 3 kinds of mutations in 5 of the examined families. Changes of c.910delC and c.876C > G were observed twice, whereas in one case, an undescribed 16-nucleotide deletion –c.867_882delGCTTGAGTACGAACCG – was detected (Figure 2). Single sequence changes were detected in exons 5 (c.733C > T), 6 (c.790_793delTTTG) and 8 (c.1010_1011delTG).

**Table 2:** List of small mutations detected in the course of the study

**Exon**	**Nucleotide change**	**Codon**	**Aminoacid change**	**Number of families**
1	c.157_158dupG [16]	53	p.Asp53Glyfsx110	1
	c.160_161insC*	54	p.Leu54ProfsX109	1
	c.178delT*	60	p.Tyr60ThrfsX 4	1
	c.180C > G [17]	60	p.Tyr60X	1
2	c.291-1G > C [18]		Splice site mutation	1
4	c.474delT*	158	p.Cys158CysfsX3	1
	c.481A > T*	161	p.Ile161Phe	1
	c.488G > A [19]	163	p.Gly163Asp	1
	c.493G > T [19]	165	p.Glu165X	1
	580G > A [20]	194	p.Asp194Asn	3
5	c.733C > T*	245	p.Leu245Phe	1
6	c.735-10C > A [19]		Possible splice site mutation	1
	c.790_793delTTTG [21]	264	p.Phe264ArgfsX22	1
7	c.876C > G [22]	292	p.Tyr292X	2
	c.867_882delGCTTGAGTACGAACCG*	289	p.Met289MetfsX42	1
	c.910delC [19]	304	p.Arg304GlyfsX32	2
8	c.921-4_921-2delGCA*		Splice site mutation	1
	c.1010_1011delTG [23]	337	p.Val337GlyfsX22	1

*novel mutation.

Apart from changes in the coding sequence, the performed investigations also made it possible to detect changes in the intron sequence embraced by the primers utilised in HRM. Before exon 2, a change c.291-1G > C was observed within the splicing site. In addition, mutations were also detected in intron 7 (c.921-4_921-2delGCA). The functional consequences of the c.735 10C > A change in intron 5 are unclear. Bioinformatic analysis performed with the SpliceView (http://zeus2.itb.cnr.it) software and the algorithm available from the University of California Berkeley website (http://www.fruitfly.org/seq_tools/splice.html) showed the potential creation of a new acceptor splicing site (scores 90 and 0.99 respectively). However, in order to confirm this hypothesis further investigations on the patient’s mRNA should be carried out.

The sequencing of the entire *STK11* coding sequence for patients in whom no evident pathogenic mutations were detected in the course of the HRM analysis failed to detect additional changes.

## Discussion

High resolution melting provides a rapid and effective screening tool for unknown mutations. This method allows to detect not only small mutations such as insertions, duplications or deletions of one or several nucleotides but also substitutions (including class 3 SNPs, e.g. c.876C > G and class 4 e.g. c.481A > T difficult to detect). In the same group of patients the mutations: c.481A > T, c.876C > G, c.180C > G, c.157-158dupG, c.735-10C > A were not detected during previous studies using Single Strand Conformation Polymorphism (SSCP) and Heteroduplex analysis (HA) (unpublished results). Thereby, the use of HRM as a screening method allowed us to increase the detection efficiency in our group of patients mainly by improving the detectability of mutations from classes 3 and 4.

In the studies of other populations the most common method used for the detection of small mutations in PJS patients is direct sequencing [8,17,22,24,25]. Only in few studies Denaturing High Performance Liquid Chromatography (DHPLC) [8] or older screening techniques such as DGGE, SSCP and HA were also used [26,27]. Depending on the studied group and the methodology used, the detection efficiency of small mutations in the above-mentioned groups is in the range from 42.42% to 70%. Therefore, the level of detection in our study with the use of HRM (54%) does not differ from the level of small mutations detection obtained using direct sequencing or other screening methods. It should be taken into account that in contrast to direct sequencing the use of screening methods allows to considerably lower the costs of analysis by reducing the number of samples subjected to sequencing. On the other hand, the advantage of HRM over other screening methods is its simplicity of implementation. Moreover, time required to perform the analysis, in comparison with other screening methods, also speaks in favor of HRM. The entire analysis, including the preparation of the reaction mixture, amplification and melting of the products takes a little more than 2 hours. The Rotor-Gene Q, utilising the rotor for 0.1 ml tubes, makes it possible to analyze during this time up to 72 amplicons simultaneously. In addition, none of the reagents present in the mixture interferes with the sequencing reaction and, therefore, it is possible to conduct sequencing directly from products previously analyzed by HRM.

Among other advantages of the HRM analysis is its single-step way of reaction preparation. Both the amplification itself as well as the subsequent fragment melting takes place in a closed system of an identical composition of the reaction mixture. No reagents need to be added in the course of the analysis which reduces the risk of sample contamination and falsification of the obtained results. In addition, the presence of the fluorescent dye throughout the duration of the reaction makes it possible to monitor not only the melting process but also the PCR itself. This opens up possibilities for designing in the future of a test detecting small mutations (HRM) combined with a simultaneous quantitative analysis on the basis of amplification curves (qPCR). The approach combining the qPCR with the HRM analysis has been already proposed by Rouleau et al. for testing the *MLH1* gene [28]. However, the qPCR analysis requires the use of a significant number of samples with known parameters to create a standard curve for each amplicon. In addition, each sample should be analyzed in replicates to properly quantify target amplicons. These two aspects significantly increase the costs of the analysis. The qPCR-HRM approach seems to be suitable and beneficial only for a large series of samples and it should be taken into account that PJS is a rare disease and usually only few samples are analyzed at the same time.

The key issue in the HRM analysis is the appropriate selection of primers for the amplification of the examined fragments so that the obtained melting profiles make it possible to observe the occurring sequence changes. In case of the *STK11* gene, high GC content reaching 63% for the entire gene sequence constitutes an additional difficulty in primer designing. Primers selected in the course of our study make it possible to obtain uniform PCR products meeting the requirements of the HRM analysis. In addition, the annealing temperature is identical for all primer pairs thus it is possible to perform screening of several exons, or even of the entire sequence coding the *STK11* gene in the course of one analysis. This is particularly important in case of investigations of such rare diseases as PJS where patient groups are small and only several samples are analyzed in the course of one examination and examination of each fragment separately would both be time-consuming and unprofitable (low cost-effectiveness).

## Conclusions

In our experiments we developed a method for a rapid detection of small mutations in the *STK11* gene in patients suffering from PJS. All mutations identified in our group of patients were detected with the assistance of the evaluated method. No additional small mutations were found in the remaining patients subjected to the sequencing of the entire sequence coding the gene. The employed HRM method exhibited 100% sensitivity in our group of patients.

## Competing interests

The authors declare that they have no competing interests.

## Authors’ contributions

PB-developed a method for a rapid detection of small mutations in the *STK11* gene, prepared the DNA samples, performed molecular analysis and drafted the manuscript. AB-prepared the DNA samples and participated in molecular analysis, TB, BN, DN, MT, JL, JL, JW, EC, MS, AK, WC and PK were responsible for PJS patients diagnosis and sample collections. PK participated in sample collection and drafted the manuscript. WC participated in coordination, and helped to draft the manuscript. AP developed the method, performed molecular analysis, drafted the manuscript and supervised all the work, conceived of the study. All authors read and approved the final manuscript.

## Pre-publication history

The pre-publication history for this paper can be accessed here:

http://www.biomedcentral.com/1471-2350/14/58/prepub
